# Cough Produced by Accumulations in the Ear

**Published:** 1880-06

**Authors:** A. E. Bridger


### COUGH PRODUCED BY ACCUMULATIONS IN THE EAR.

THE patient, a singularly robust young lady, consulted me in regard to a cough of some three years' standing. The cough was loud, incessant and peculiarly hollow. It was dry, unaffected by times of day, seasons, or weather. It deprived her often of rest at night, and rendered her a source of annoyance and anxiety to her friends. She had consulted various medical men, and had taken almost every conceivable patent medicine, including some powerful sedatives, without obtaining even slight relief. The heart and lungs proved, as I had expected, to be healthy. The functions of the uterine, gastro-intestinal, and renal systems were stated to be strictly normal. There were no symptoms indicative of the presence of entozoa. The condition of the throat was natural; there was no relaxation of the uvula. I had come to the conclusion that the cough must be of a hysterical nature, when it occurred to me to examine the ears. The left membrana tympani was plainly visible and healthy. The state of the right one was hidden by a dark mass. On touching this mass with a probe, through the speculum, the patient's peculiar cough was immediately produced, and by keeping up a very slight, steady pressure on it, a fit of coughing, not unlike a violent paroxysm in whooping cough, resulted. By the aid of a large ear-syringe and a weak, hot alkaline solution, a piece of hard wax, *fons et origo mali*, was with some difficulty produced. It weighed over three grains. I followed up the syringing by the use of Politzer's apparatus. The cough ceased, and though some weeks have now elapsed, it shows no sign of returning.—A. E. Bridger, M. B., in the *Lancet*, March 6, 1880.

